# Comment on Bokayeva et al. Vitamin Status in Patients with Phenylketonuria: A Systematic Review and Meta-Analysis. *Int. J. Mol. Sci*. 2024, *25*, 5065

**DOI:** 10.3390/ijms27052174

**Published:** 2026-02-26

**Authors:** Muhammad Iqhrammullah

**Affiliations:** Postgraduate Program of Public Health, Universitas Muhammadiyah Aceh, Banda Aceh 23245, Indonesia; m.iqhram@unmuha.ac.id

I read with great interest the work by Bokayeva et al., reporting their systematic review findings on vitamin status in patients with phenylketonuria (PKU) [1]. PKU is a genetic disorder caused by a deficiency of phenylalanine hydroxylase, necessitating lifelong restriction of natural protein to prevent excessive phenylalanine accumulation. Consequently, individuals with PKU have limited access to natural vitamin D sources such as fish, eggs, and liver, which may increase their risk of deficiency. Thus, the study by Bokayeva et al. provides a valuable overview of micronutrient status in this population [1]. However, several methodological and interpretive concerns limit the reliability of the conclusions, particularly regarding vitamin D.

The authors reported separate meta-analyses for total vitamin D and 1,25-dihydroxyvitamin D (1,25(OH)_2_D_3_), yet their inclusion criteria did not distinguish between specific metabolites or assay types [1]. Studies measuring 25(OH)D, the clinically accepted marker of vitamin D status, were pooled together with studies measuring 25(OH)D_3_, cholecalciferol, or unspecified “vitamin D” [1]. Because these metabolites reflect different physiological processes (status, intake, and active hormonal regulation), combining them in a single meta-analysis may obscure true differences and risk misleading interpretation of vitamin D status in PKU. In addition, the pooled studies also used different analytical instruments. Since these markers differ physiologically and analytically, merging them introduces measurement heterogeneity, explaining the extremely high *I*^2^ values (up to 96%) and weakening biological interpretability [1]. Furthermore, the changing composition of protein substitutes over time introduces additional heterogeneity, further complicating the meta-analysis.

The authors’ conclusion that “PKU patients have higher folate and 1,25-dihydroxyvitamin D levels compared to controls” is not appropriate [1], as it overlooks the substantial heterogeneity across studies and the limitations in metabolite selection and assay comparability, which preclude drawing a definitive directional difference. Although the authors do acknowledge uncertainty in the Discussion, this nuance is not accurately reflected in the Abstract or the formal Conclusion, where statistically significant results are mentioned without contextualizing the underlying variability in the evidence [1].

The pooled estimate for total vitamin D is heavily influenced by Nagasaka et al. (2011), a small adult study (34 PKU patients, 36 controls) reporting an unusually large effect size (SMD = −3.52). For 1,25(OH)_2_D_3_, the pooled result (standardized mean difference, SMD = 2.059, *p* = 0.026; *I*^2^ = 94.7%) was driven largely by the same research group [2,3]. Once outlier or high-risk studies were excluded, the association became non-significant (SMD = 2.768, *p* = 0.084) [1]. I re-analyzed the pooled estimates using a DerSimonian–Laird random-effects model and observed that Nagasaki et al., 2013, exceeded the threshold for Cook’s distance (1.33, derived from 4/*n*) [3]. 

Nagasaka et al. (2011) also introduced several methodological limitations. Vitamin D intake was assessed via a food-frequency questionnaire [2], thus potentially having recall bias. The study reported higher serum 1,25(OH)_2_D_3_ in PKU patients (*p* < 0.05), while 25(OH)D_3_, calcium, phosphate, and parathyroid hormone (PTH) levels were all within normal ranges and showed no between-group differences [2]. The authors speculated that the rise in 1,25(OH)_2_D_3_ “might indicate enhanced renal 1α-hydroxylase activity [2],” and although this physiological possibility cannot be excluded, the available data provide no biochemical markers to substantiate altered mineral or endocrine regulation. Given these limitations, such mechanistic interpretations should be framed cautiously and regarded as hypotheses rather than definitive explanations supported by the evidence, thereby highlighting an important opportunity for future studies to investigate these pathways more directly.

I acknowledge that the study by Bokayeva et al. is a systematic review and meta-analysis [1], which relies on aggregated data and is not designed to establish mechanistic pathways. However, this makes careful statistical interpretation even more essential. The reported differences in vitamin D metabolites must be contextualized within the substantial uncertainty of the evidence. The conclusion should emphasize uncertainty rather than significance, with a clearer distinction between vitamin D metabolites and more cautious attribution of potential compensatory mechanisms.

## Abbreviations

The following abbreviations are used in this manuscript:

1,25(OH)_2_D_3_	1,25-Dihydroxyvitamin D
PKU	Patients with phenylketonuria
SMD	Standardized mean difference
PTH	Parathyroid hormone
